# Cesarean section and parenting stress: Results from the Japan Environment and Children’s Study

**DOI:** 10.1192/j.eurpsy.2023.5

**Published:** 2023-01-24

**Authors:** Kenta Matsumura, Takehiro Hatakeyama, Taketoshi Yoshida, Akiko Tsuchida, Hidekuni Inadera, The Japan Environment and Children’s Study (JECS) Group

**Affiliations:** 1 Department of Public Health, Faculty of Medicine, University of Toyama, Toyama, Japan; 2 Toyama Regional Center for JECS, University of Toyama, Toyama, Japan; 3 Division of Neonatology, Maternal and Perinatal Center, Toyama University Hospital, Toyama, Japan

**Keywords:** Epidemiology, longitudinal study, mode of delivery, pain, stress

## Abstract

**Background:**

Accumulating evidence suggests a long-term health risk of cesarean section for the mother and child, but few studies have examined the link between cesarean section and parenting stress. Here, we examined this association by exploiting a large dataset.

**Methods:**

Participants were 65,235 mothers participating in the Japan Environment and Children’s Study, an ongoing nationwide birth cohort. Outcome variables were parenting stress assessed as total score and subscale scores (representing the difficult child, parental distress, and spouse factors) on the Japanese 19-item version of the Parenting Stress Index Short Form (J-PSI-SF). Exposures were the mode of delivery, the timing of the J-PSI-SF assessment (1.5, 2.5, and 3.5 years postpartum), and the interaction between them. Multivariate regression analysis was used to calculate adjusted *β* coefficients and standard error of the means (SEMs).

**Results:**

The J-PSI-SF total score was higher in the cesarean section group than in the vaginal delivery group (adjusted *β* = 0.24, SEM = 0.09). This increase was primarily due to higher scores for the difficult child factor (adjusted *β* = 0.18, SEM = 0.05) and not to higher scores for the parental distress or spouse factor.

**Conclusions:**

Cesarean section was associated with higher parenting stress, especially in relation to the difficult child factor. Our results highlight the importance of paying particular attention to the mental health of both mother and child in the case of cesarean section.

## Introduction

Parenting stress is stress that occurs when the parenting demands from the child exceed the parenting resources of the parents [1]. Parenting stress can be broadly classified into stress related to parent and child factors. Increased parenting stress can lead to (1) increased risk of child maltreatment [2, 3], (2) poor parenting behavior [4], (3) lower quality of life for parents [5], (4) worse communication within the family [6], and (5) increased risk of emotional and behavioral problems in the child [7]. Therefore, increased parental stress could be a significant public health concern.

One factor that potentially increases parenting stress is cesarean section. The number of cesarean sections continues to grow worldwide [8], and a large observational study has reported increased parenting stress associated with cesarean sections [9]. Although the actual mechanism behind the association is not known, the prolonged physical pain resulting from a cesarean section may have some involvement. Compared with vaginal delivery, cesarean section is associated with higher pain even years after delivery [10, 11], and this pain may reduce the mother’s parenting resources available to cope with the demands of childcare. Childhood illness may also have some involvement. It has been reported that cesarean sections are associated with childhood asthma, obesity [12, 13], and stress-related disorders [14], which may make a child more demanding. However, the relationship between cesarean section and parenting stress is still not well understood, nor whether this relationship can be attributed to the mother herself, the child, or both.

In this study, we sought to determine the relationship between cesarean delivery and parenting stress in the Japan Environment and Children’s Study (JECS), a large birth cohort study in Japan.

## Methods

### Study design and population

Participants were mothers enrolled in JECS, an ongoing nationwide government-funded birth cohort study examining the impact of various environmental factors on children’s health and development. The design and baseline characteristics of the JECS have been reported in detail elsewhere [15, 16]. Briefly, pregnant women were enrolled between January 2011 and March 2014 at public facilities, such as health centers and obstetrics and gynecology departments, from 15 regional centers (in rural and urban locations) across Japan via face-to-face recruitment. Follow-up took place during mid/late pregnancy, at childbirth, and at 1.5, 2.5, and 3.5 years postpartum. Data were collected from the medical record transcriptions by physicians, midwives/nurses, and/or research coordinators and via self-report questionnaires distributed to the participants by hand at the cooperating health care providers during pregnancy and by post after childbirth.

**Figure 1.**
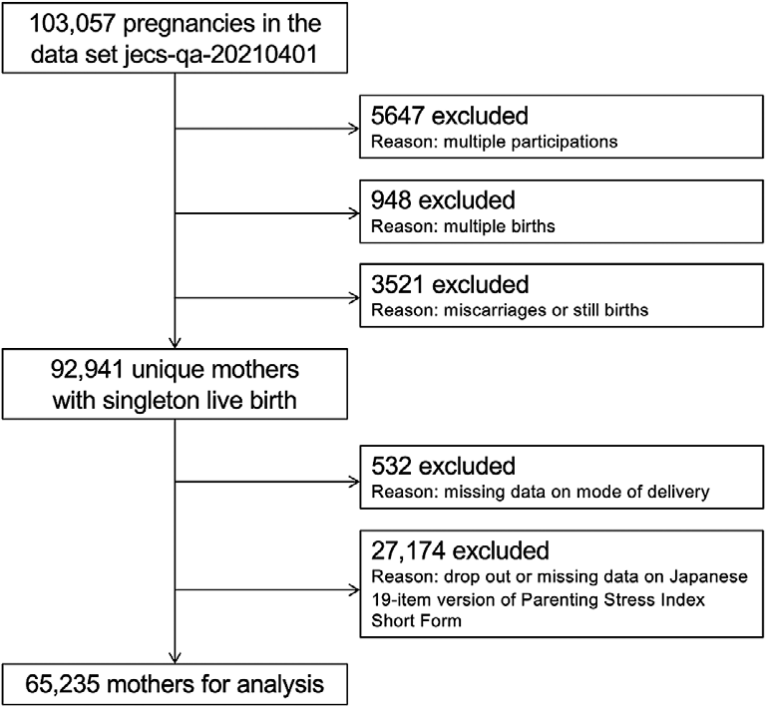
Participant flow diagram.

This study analyzed the jecs-qa-20210401 dataset, which was first released in April 2021 and completed in February 2022. The dataset contains data on 103,057 pregnancies. In this study, 5,647 pregnancies were excluded due to multiple registrations (second or third registration of the same mother), 949 due to multiple births (twins or more), and 3,562 due to miscarriages or stillbirths. Among the remaining 92,941 unique mothers, an additional 532 were excluded due to dropout or missing data on mode of delivery, and another 27,174 were excluded due to dropout or missing data on parenting stress. This left 65,235 mothers for the final analysis (Figure 1).

All procedures contributing to this work complied with the ethical standards of the relevant national and institutional committees on human experimentation and with the Helsinki Declaration of 1975, as revised in 2008. The JECS protocol was reviewed and approved by the Ministry of the Environment’s Institutional Review Board on Epidemiological Studies (100910001) and the ethics committees of all participating institutions. Written informed consent was obtained from all participants. The protocol of this study was also approved by the Ethics Committee of the University of Toyama (Protocol number R2022091).

### Measures

#### Primary exposure

Mode of delivery (cesarean section or vaginal delivery), obtained from the medical record transcriptions, was used as the primary exposure variable.

#### Outcomes

Parenting stress was assessed on three occasions, at 1.5, 2.5, and 3.5 years postpartum, using the Japanese 19-item version of the Parenting Stress Index Short Form (J-PSI-SF) [17]. This instrument was developed to evaluate postpartum parenting stress based on the 78-item PSI [18], which had itself originated from Abidin’s 101-item PSI [1, 19]. The J-PSI-SF has good internal consistency (Cronbach α = 0.87), test–retest consistency over a 1-year interval (*r* = 0.73), and a three-factor structure composed of difficult child, parental distress, and spouse factors [20] (Supplementary Table S1).

The difficult child factor is defined as stress from the child’s temperament or behavior and consists of items 3, 4, 6, 8, 9, 10, and 11, including “10. My child imposes demands on me more than most children,” “9. My child is easily upset by small things,” and “6. My child cries or fusses rather often.”

The parental distress factor is defined as the stress felt by the parent while raising the child and consists of items 1, 2, 12, 13, 14, 17, 18, and 19, including “19. I am unable to enjoy things as I used to,” “12. I do not feel capable of handling things very well,” and “17. I feel alone without friends.”

The spouse factor is defined as stress in the relationship with the spouse/partner while raising the child and consists of two items, “16. There are more problems with my spouse by having a child” and “15. My spouse does not give me as much help as I expected.”

Mothers were asked to complete their response on a 5-point Likert scale (1 = strongly disagree, 2 = disagree, 3 = not sure, 4 = agree, and 5 = strongly agree), and items with reversed valence were scored by reversing scores 1 to 5. We calculated the subscale scores for the difficult child, parental distress, and spouse factors by summing the scores of corresponding items and the total J-PSI-SF score by summing all subscale scores and then used them as outcome variables.

#### Potential confounders

We selected potential confounders, defined as variables before and/or during pregnancy with a potential impact on both exposures and outcomes, as well as basic anthropometric and socioeconomic variables. These variables were maternal age during pregnancy, pre-pregnancy body mass index (BMI), parity, gestational age, pregnancy complication (e.g., hypertension, diabetes, and psychiatric disease), obstetrics complication (e.g., placenta previa, placental abruption, and intrauterine growth restriction), marital status, highest education level [21], employment status, annual household income, smoking status, alcohol intake, regular physical activity corresponding to a 10-min walk/day [22, 23], experience of a stressful event, negative attitude toward pregnancy, degree of emotional support [24], and psychological distress assessed using the Kessler 6 (K6) questionnaire [25–27], and area of residence (regional center where participants were recruited). Variables were categorized according to standard medical practice or common practice in Japan (e.g., [28]).

### Statistical analysis

Descriptive characteristics are presented as frequencies and percentages. Multivariate linear regression analysis was performed to estimate crude and adjusted *β* coefficients and the standard errors of the means (SEMs). The exposure variables were the mode of delivery, timing of the J-PSI-SF assessment in the postpartum period (repeated measures), and the interaction between mode of delivery and timing of the J-PSI-SF assessment. Vaginal delivery and assessment at 1.5 years were used as the reference. Outcome variables were each score derived from the J-PSI-SF. The forced entry method was used to include all the potential confounders and their interactions with assessment timing to adjust the model.

Data were analyzed using SAS version 9.4 software (SAS Institute Inc., Cary, North Carolina).

#### Additional analysis

For a more in-depth analysis, we repeated the regression analysis using each item score of the J-PSI-SF.

## Results

In total, 65,235 mothers were analyzed; the mean age ± standard deviation (SD) was 31.4 ± 4.8 years, and the mean pre-pregnancy BMI ± SD was 21.1 ± 3.2. Table 1 shows the participants’ characteristics according to the mode of delivery. Among 65,235 mothers, 12,049 had a cesarean section and 53,186 had a vaginal delivery. Cesarean section varied from 14.1 to 24.9% according to area of residence.

**Table 1.** Characteristics of participants according to mode of delivery.

	Vaginal delivery		Cesarean section	
	(*n* = 53,186)		(*n* = 12,049)	
	*n*	(%)	*n*	(%)
Age, y				
<25	4486	(8.4)	609	(5.1)
25–<30	15,578	(29.3)	2493	(20.7)
30–<35	19,473	(36.6)	4249	(35.3)
≥35	13,421	(25.2)	4615	(38.3)
Missing	228	(0.4)	83	(0.7)
Body mass index, kg/m^2^				
<18.5	9068	(17.1)	1521	(12.6)
18.5–<25	39,669	(74.6)	8699	(72.2)
≥25	4418	(8.3)	1819	(15.1)
Missing	31	(0.1)	10	(0.1)
Parity				
0	22,792	(42.9)	5192	(43.1)
1	19,264	(36.2)	4492	(37.3)
≥2	9792	(18.4)	2057	(17.1)
Missing	1338	(2.5)	308	(2.6)
Gestational age, week				
<34	178	(0.3)	407	(3.4)
34–<37	1484	(2.8)	863	(7.2)
37–<42	51,424	(96.7)	10,716	(88.9)
≥42	100	(0.2)	63	(0.5)
Pregnancy complication				
No	44,920	(84.5)	9434	(78.3)
Yes	7310	(13.7)	2380	(19.8)
Missing	956	(1.8)	235	(2.0)
Obstetric complications				
No	29,095	(54.7)	5415	(44.9)
Yes	23,187	(43.6)	6402	(53.1)
Missing	904	(1.7)	232	(1.9)
Marital status				
Married	51,073	(96.0)	11,621	(96.5)
Unmarried	1453	(2.7)	278	(2.3)
Divorced/widowed	241	(0.5)	75	(0.6)
Missing	419	(0.8)	75	(0.6)
Highest education level, y				
≤9	1657	(3.1)	397	(3.3)
>9–≤12	15,254	(28.7)	3604	(29.9)
>12–≤16	22,913	(43.1)	5263	(43.7)
>16	12,914	(24.3)	2651	(22.0)
Missing	448	(0.8)	134	(1.1)
Employment status				
No	23,971	(45.1)	5467	(45.4)
Yes	28,623	(53.8)	6408	(53.2)
Missing	592	(1.1)	174	(1.4)
Annual household income, JPY				
<4 million	18,849	(35.4)	4158	(34.5)
4–<6 million	16,975	(31.9)	3772	(31.3)
≥6 million	13,929	(26.2)	3329	(27.6)
Missing	3433	(6.5)	790	(6.6)
Smoking status				
Never	32,566	(61.2)	7036	(58.4)
Quit	18,445	(34.7)	4475	(37.1)
Current	1618	(3.0)	390	(3.2)
Missing	557	(1.1)	148	(1.2)
Alcohol intake				
Never	17,934	(33.7)	3999	(33.2)
Former	33,280	(62.6)	7592	(63.0)
Current	1391	(2.6)	294	(2.4)
Missing	581	(1.1)	164	(1.4)
Regular physical activity				
No	12,377	(23.3)	3072	(25.5)
Yes	40,473	(76.1)	8869	(73.6)
Missing	336	(0.6)	108	(0.9)
Experience of stressful event				
No	29,957	(56.3)	6811	(56.5)
Yes	22,695	(42.7)	5092	(42.3)
Missing	534	(1.0)	146	(1.2)
Negative attitude toward pregnancy				
No	49,165	(92.4)	11,246	(93.3)
Yes	3578	(6.7)	723	(6.0)
Missing	443	(0.8)	80	(0.7)
Degree of emotional social support				
Low	14,454	(27.2)	3354	(27.8)
Mid–low	10,798	(20.3)	2440	(20.3)
Mid–high	14,422	(27.1)	3209	(26.6)
High	12,525	(23.6)	2800	(23.2)
Missing	987	(1.9)	246	(2.0)
Kessler psychological distress scale (K6) score				
0–4	38,558	(72.5)	8776	(72.8)
5–12	12,958	(24.4)	2821	(23.4)
≥13	1339	(2.5)	348	(2.9)
Missing	331	(0.6)	104	(0.9)
Area of residence				
A	4160	(7.8)	1019	(8.5)
B	4627	(8.7)	944	(7.8)
C	6822	(12.8)	1567	(13.0)
D	2964	(5.6)	724	(6.0)
E	3730	(7.0)	613	(5.1)
F	3761	(7.1)	766	(6.4)
G	3223	(6.1)	617	(5.1)
H	3017	(5.7)	681	(5.7)
I	2218	(4.2)	535	(4.4)
J	4123	(7.8)	978	(8.1)
K	2724	(5.1)	654	(5.4)
L	1668	(3.1)	376	(3.1)
M	3153	(5.9)	1046	(8.7)
N	4223	(7.9)	849	(7.1)
O	2773	(5.2)	680	(5.6)

Abbreviation: K6, Kessler psychological distress scale.

Table 2 shows unstandardized crude and adjusted *β* coefficients (SEMs) for each J-PSI-SF score according to mode of delivery, timing of J-PSI-SF assessment, and their interaction. The J-PSI-SF total score was higher in the cesarean section group than in the vaginal delivery group (adjusted *β* = 0.24, SEM = 0.09). This increase was almost entirely due to an increase in the difficult child factor score (adjusted *β* = 0.18, SEM = 0.05) and not to the parental distress factor score (adjusted *β* = 0.07, SEM = 0.04) or spouse factor score (adjusted *β* = −0.01, SEM = 0.02). The total score was higher at 2.5 years postpartum (adjusted *β* = 0.57, SEM = 0.23) than at 1.5 years postpartum. This increase was mainly due to an increase in the parental distress factor score (adjusted *β* = 0.43, SEM = 0.12). The total score was somewhat lower at 3.5 years postpartum (adjusted *β* = −0.46, SEM = 0.26) than at 1.5 years postpartum, owing to the combination of a large decrease in the difficult child factor score (adjusted *β* = −0.54, SEM = 0.15) and a small increase in the parental distress factor score (adjusted *β* = 0.19, SEM = 0.13). No significant interaction was observed between cesarean section and timing of assessment.

**Table 2.** Unstandardized crude and adjusted *β* coefficients (SEMs) for each score of the Japanese 19-item version of the Parenting Stress Index Short Form according to mode of delivery and timing of assessment in the postpartum period.

	Effect							
	Main					Interaction		
	Cesarean section		Timing			Cesarean section × Timing		
	Yes	No	2.5 y	3.5 y	1.5 y	Yes × 2.5 y	Yes × 3.5 y	Others
*Crude model*								
Total score	**0.39 (0.09)**	—	**0.44 (0.03)**	**−0.54 (0.03)**	—	0.00 (0.07)	−0.06 (0.08)	—
Subscale score								
Difficult child	**0.21 (0.05)**	—	**0.09 (0.02)**	**−0.55 (0.02)**	—	0.02 (0.04)	0.00 (0.05)	—
Parental distress	**0.18 (0.05)**	—	**0.31 (0.02)**	0.03 (0.02)	—	−0.02 (0.03)	−0.06 (0.04)	—
Spouse	0.00 (0.02)	—	**0.04 (0.01)**	−0.01 (0.01)	—	0.01 (0.02)	0.00 (0.02)	—
*Adjusted model* ^a^								
Total score	**0.24 (0.09)**	—	**0.57 (0.23)**	−0.46 (0.26)	—	−0.05 (0.07)	−0.13 (0.08)	—
Subscale score								
Difficult child	**0.18 (0.05)**	—	0.11 (0.14)	**−0.54 (0.15)**	—	−0.02 (0.04)	−0.04 (0.05)	—
Parental distress	0.07 (0.04)	—	**0.43 (0.12)**	0.19 (0.13)	—	−0.03 (0.04)	−0.07 (0.04)	—
Spouse	−0.01 (0.02)	—	0.03 (0.06)	−0.10 (0.06)	—	−0.01 (0.02)	−0.01 (0.02)	—

*Note*: Boldface type indicates significance, defined as the 95% confidence interval not crossing the reference (=0.00). “—” represents reference. Others include Yes × 1.5 y, No × 1.5 y, No × 2.5 y, and No × 3.5 y.

Abbreviation: SEM, standard error of the mean.

a Adjusted for maternal age, body mass index, parity, gestational age, pregnancy complication, obstetric complication marital status, highest education level, employment status, annual household income, smoking status, alcohol intake, regular physical activity, experience of stressful event, negative attitude toward pregnancy, emotional social support, psychological distress, and area of residence.

Table 3 summarizes the results of the additional analysis. As in the main analysis, the additional analysis using each J-PSI-SF item revealed that cesarean section was associated with higher scores on items 3, 4, 6, 10, and 11 for the difficult child factor. Additionally, cesarean section was associated with higher scores only on item 18 for the parental distress factor and item 5, which does not belong to any factor. Again, no significant interaction was observed between cesarean section and timing of assessment.

**Table 3.** Unstandardized adjusted *β* coefficients (SEMs)^a^ for each item of the Japanese 19-item version of the Parenting Stress Index Short Form according to mode of delivery and timing of assessment in the postpartum period.

Item	Factor		Effect							
			Main					Interaction		
			Cesarean section		Timing			Cesarean section × Timing		
			Yes	No	2.5 y	3.5 y	1.5 y	Yes × 2.5 y	Yes × 3.5 y	Others
1	PD	I enjoy being a parent	0.007 (0.007)	—	**0.078 (0.022)**	**0.159 (0.023)**	—	0.003 (0.007)	−0.005 (0.007)	—
2	PD	When caring for my child is difficult, I resort to asking for help or advice	0.005 (0.008)	—	0.020 (0.026)	**0.068 (0.027)**	—	−0.010 (0.008)	−0.008 (0.008)	—
3	DC	My child is active to the extent of overwhelming me	**0.038 (0.011)**	—	**0.099 (0.035)**	0.037 (0.037)	—	−0.013 (0.011)	−0.017 (0.012)	—
4	DC	My child has difficulty focusing attention	**0.026 (0.009)**	—	0.020 (0.032)	−0.028 (0.034)	—	0.005 (0.010)	−0.003 (0.011)	—
5	UI	My child rarely does things that make me feel good	**0.025 (0.006)**	—	0.017 (0.022)	**0.050 (0.023)**	—	−0.010 (0.007)	−0.012 (0.007)	—
6	DC	My child cries or fusses rather often	**0.028 (0.009)**	—	0.021 (0.033)	**−0.078 (0.034)**	—	−0.018 (0.010)	−0.018 (0.010)	—
7	UI	My child does not smile very much, unlike most children	0.006 (0.005)	—	−0.011 (0.016)	**−0.039 (0.017)**	—	0.004 (0.005)	−0.002 (0.005)	—
8	DC	My child does some things that bother me a lot	0.011 (0.011)	—	0.041 (0.039)	−0.012 (0.040)	—	0.001 (0.012)	−0.001 (0.013)	—
9	DC	My child is easily upset by small things	0.011 (0.011)	—	−0.007 (0.037)	−0.074 (0.039)	—	0.002 (0.011)	−0.017 (0.012)	—
10	DC	My child imposes demands on me more than most children	**0.027 (0.010)**	—	**0.145 (0.033)**	**0.126 (0.037)**	—	−0.001 (0.010)	0.009 (0.011)	—
11	DC	My child is always attached to me	**0.035 (0.011)**	—	**−0.213 (0.037)**	**−0.514 (0.040)**	—	0.007 (0.012)	0.002 (0.012)	—
12	PD	I do not feel able to handle things very well	0.007 (0.010)	—	**0.081 (0.031)**	0.026 (0.033)	—	−0.005 (0.009)	−0.014 (0.010)	—
13	PD	Since giving birth to my child, I feel unable to do most of the things I like to do	0.004 (0.010)	—	**−0.065 (0.032)**	**−0.182 (0.033)**	—	−0.005 (0.010)	0.002 (0.010)	—
14	PD	I feel that it is my fault every time my child does something wrong	−0.002 (0.009)	—	**0.065 (0.030)**	0.046 (0.031)	—	−0.012 (0.009)	−0.016 (0.010)	—
15	SP	My spouse does not give me as much help as I expected	−0.007 (0.011)	—	0.041 (0.032)	−0.020 (0.036)	—	−0.001 (0.010)	−0.008 (0.010)	—
16	SP	Having a child has created more problems with my spouse	−0.002 (0.011)	—	−0.007 (0.034)	**−0.085 (0.036)**	—	−0.009 (0.010)	−0.003 (0.011)	—
17	PD	I feel alone with no friends	−0.004 (0.009)	—	0.012 (0.026)	−0.023 (0.027)	—	0.007 (0.008)	−0.009 (0.008)	—
18	PD	I have been experiencing more illness, aches, and pains over the past 6 months	**0.036 (0.010)**	—	**0.148 (0.037)**	0.047 (0.038)	—	−0.008 (0.011)	−0.016 (0.012)	—
19	PD	I am unable to enjoy things as I used to	0.015 (0.008)	—	**0.088 (0.028)**	0.044 (0.030)	—	0.003 (0.009)	−0.005 (0.009)	—

*Note:* Boldface type indicates significance, defined as the 95% confidence interval not crossing the reference (=0.00). “—” represents reference. Others include Yes × 1.5 y, No × 1.5 y, No × 2.5 y, and No × 3.5 y.

Abbreviations: DC, difficult child; PD, parental distress; SEM, standard error of the mean; SP, spouse; UI, unused item not belonging to any factor.

a Adjusted for maternal age, body mass index, parity, gestational age, pregnancy complication, obstetric complication marital status, highest education level, employment status, annual household income, smoking status, alcohol intake, regular physical activity, experience of stressful event, negative attitude toward pregnancy, emotional social support, psychological distress, and area of residence.

## Discussion

This study examined the relationship between cesarean section and parenting stress. The results consistently showed a slight, but significant, increase in J-PSI-SF total scores for the cesarean section group from as early as 1.5 years of age. This increase was primarily due to an increase in the J-PSI-SF difficult child factor score (i.e., stress from the child’s temperament or behavior), and not in the score for factors related to parental distress (i.e., stress felt by the mother during childrearing) or spouse (i.e., stress in the relationship with spouse while raising the child). The finding of increased parenting stress associated with a cesarean section is consistent with the results of a previous large cohort study [9].

The J-PSI-SF difficult child factor score was higher for children delivered by cesarean section. One reason for this may be that allergic disease and obesity are more common in these children [12, 13]. Studies of parenting stress have consistently found that parenting stress is increased when a child has an autism spectrum disorder, allergy, or physical disease [19]. Our previous study also showed an increased prevalence of functional constipation in children born by cesarean section [29], which also likely increases the parenting demands from the child.

In relation to the above, children born by cesarean section are known to exhibit an excessive stress response to acute psychological stressors even in adulthood [30]. It has been suggested that passage through the birth canal is critical in the development of the core stress system, namely, the hypothalamic–pituitary–adrenal axis, and bypassing this may have a negative impact on strong stress experiences [31]. It has also been reported that children born by cesarean sections have low diversity of gut microbiota, and such low diversity is related to psychiatric disorders [32]. Recent epidemiological studies have reported that the only factor associated with stress-related disorders after controlling for siblings is cesarean section [14], and cesarean section was associated with neurodevelopmental disorders when not controlling for siblings [33]. Further research is needed to investigate the impact of cesarean section on psychiatric disorders, neurodevelopment disorders, and stress reactions in children, as well as allergy and obesity issues, all of which are highly likely to lead to a more demanding child.

When analyzed on an individual item basis, cesarean section was found to be associated with many of the items comprising the J-PSI-SF difficult child factor, consistent with our main results. More specifically, children born by cesarean section had worse scores on items for being too active, having difficulty concentrating, not pleasing their mother, being prone to crying and fussing, being demanding, and being attached to the mother. These responses are suggestive of attention deficit hyperactivity disorder, hyperarousal, separation anxiety, and being prone to illness. As noted in the introduction, the following pathway can be posited: (1) cesarean section; (2) neuropsychiatric and developmental disorders and other illnesses; and (3) increased J-PSI-SF difficult child factor scores. For the parental distress factor, on the other hand, the only significant association observed was between cesarean section and illness, aches, and pains, and there was no association with other items such as not enjoying being a parent, having less social support, or feeling unable to cope with things. This association between cesarean section and pain is consistent with previous studies [10, 11], but this alone would not have led to a clear reduction in parenting resources, and thus was not associated with J-PSI-SF parental distress factor score as the umbrella factor.

Cesarean sections can be a life-saving procedure when medically indicated, but they are increasing worldwide [8], and Japan is no exception [34]. In particular, the number of procedures performed at the request of the mother is increasing [35]. Generally, it is known that cesarean sections not only increase maternal mortality and morbidity, but also cause allergic diseases and obesity in the child. Additionally, recent large epidemiological studies have shown that cesarean sections increase the risk of psychiatric disorders [14]. Therefore, taken together with the results of this study, the decision to have a cesarean section should not be made lightly.

This study has the following strengths. First, we analyzed data from an ongoing nationwide birth cohort with a large sample exceeding 65,000 mothers who were recruited from both urban and rural areas between 2011 and 2014. Therefore, the sample can be considered to be representative of recent Japanese mothers. Second, assessments were performed three times, at 1.5, 2.5, and 3.5 years postpartum. This allowed us to obtain rich results. Third, we considered 16 potential confounders, so our estimates are likely reasonable.

This study also has some limitations. First, we derived data on the delivery mode from the medical record transcriptions, but we have no data on whether the cesarean section was elective (scheduled) or emergency. If we had distinguished between these, the results might have been slightly different, given that these differences in caesarian section are reported to affect the mental state of mothers [36] and neurodevelopmental disorders when not controlling for siblings [33]. Second, mothers with high parenting stress may have dropped out of the cohort, possibly resulting in selection bias. Finally, the generalizability of the association found in this study must be examined in other settings outside Japan, where the J-PSI-SF has not yet been validated, and in other populations in addition to mothers.

In conclusion, this study found that cesarean section was associated with increased parenting stress, especially in terms of the difficult child factor, but not with the parental distress or spouse factor. In the case of cesarean section, particular attention should be paid to the mental health of both mother and child.

## Data Availability

Data are unsuitable for public deposition due to ethical restrictions and the legal framework of Japan. It is prohibited by the Act on the Protection of Personal Information (Act No. 57 of 30 May 2003, amendment September 9, 2015) to publicly deposit the data containing personal information. Ethical Guidelines for Medical and Health Research Involving Human Subjects enforced by the Japanese Ministry of Education, Culture, Sports, Science and Technology and the Ministry of Health, Labour and Welfare also restrict the open sharing of the epidemiologic data. All inquiries about access to data should be sent to: jecs-en@nies.go.jp. The person responsible for handling inquiries sent to this e-mail address is Dr Shoji F. Nakayama, JECS Programme Office, National Institute for Environmental Studies.

## Supplementary material

For supplementary material accompanying this paper visit http://doi.org/10.1192/j.eurpsy.2023.5. click here to view supplementary material

## A. Appendix

Members of the JECS Group as of 2022: Michihiro Kamijima (Principal Investigator, Nagoya City University, Nagoya, Japan), Shin Yamazaki (National Institute for Environmental Studies, Tsukuba, Japan), Yukihiro Ohya (National Center for Child Health and Development, Tokyo, Japan), Reiko Kishi (Hokkaido University, Sapporo, Japan), Nobuo Yaegashi (Tohoku University, Sendai, Japan), Koichi Hashimoto (Fukushima Medical University, Fukushima, Japan), Chisato Mori (Chiba University, Chiba, Japan), Shuichi Ito (Yokohama City University, Yokohama, Japan), Zentaro Yamagata (University of Yamanashi, Chuo, Japan), Hidekuni Inadera (University of Toyama, Toyama, Japan), Takeo Nakayama (Kyoto University, Kyoto, Japan), Tomotaka Sobue (Osaka University, Suita, Japan), Masayuki Shima (Hyogo Medical University, Nishinomiya, Japan), Hiroshige Nakamura (Tottori University, Yonago, Japan), Narufumi Suganuma (Kochi University, Nankoku, Japan), Koichi Kusuhara (University of Occupational and Environmental Health, Kitakyushu, Japan), and Takahiko Katoh (Kumamoto University, Kumamoto, Japan).

## References

[1] Abidin RR . Parenting stress index. 3rd ed. Odessa: Psychological Assessment Resource; 1995.

[2] Rodriguez CM , Green AJ . Parenting stress and anger expression as predictors of child abuse potential. Child Abuse Negl. 1997;21:367–77. 9134265 10.1016/s0145-2134(96)00177-9

[3] Rodriguez CM , Richardson MJ . Stress and anger as contextual factors and preexisting cognitive schemas: predicting parental child maltreatment risk. Child Maltreat. 2007;12:325–37. 17954939 10.1177/1077559507305993

[4] Gerdes AC , Hoza B , Arnold LE , Pelham WE , Swanson JM , Wigal T , et al. Maternal depressive symptomatology and parenting behavior: exploration of possible mediators. J Abnorm Child Psychol. 2007;35:705–14. 17674187 10.1007/s10802-007-9134-3

[5] Cho KS , Hong EJ . A path analysis of the variables related to the quality of life of mothers with disabled children in Korea. Stress Health. 2013;29:229–39. 23023819 10.1002/smi.2457

[6] Ponnet K , Wouters E , Mortelmans D , Pasteels I , De Backer C , Van Leeuwen K , et al. The influence of mothers’ and fathers’ parenting stress and depressive symptoms on own and partner’s parent-child communication. Fam Process. 2013;52:312–24. 23763689 10.1111/famp.12001

[7] Bakoula C , Kolaitis G , Veltsista A , Gika A , Chrousos GP . Parental stress affects the emotions and behaviour of children up to adolescence: a Greek prospective, longitudinal study. Stress. 2009;12:486–98. 19206015 10.3109/10253890802645041

[8] Betran AP , Ye J , Moller AB , Zhang J , Gulmezoglu AM , Torloni MR . The increasing trend in caesarean section rates: global, regional and national estimates: 1990-2014. PLoS One. 2016;11:e0148343. 26849801 10.1371/journal.pone.0148343 PMC4743929

[9] Chen HH , Lai JC , Hwang SJ , Huang N , Chou YJ , Chien LY . Understanding the relationship between cesarean birth and stress, anxiety, and depression after childbirth: a nationwide cohort study. Birth. 2017;44:369–76. 28594092 10.1111/birt.12295

[10] Kainu JP , Sarvela J , Tiippana E , Halmesmaki E , Korttila KT . Persistent pain after caesarean section and vaginal birth: a cohort study. Int J Obstet Anesth. 2010;19:4–9. 19733050 10.1016/j.ijoa.2009.03.013

[11] Li WY , Liabsuetrakul T , Stray-Pedersen B , Li YJ , Guo LJ , Qin WZ . The effects of mode of delivery and time since birth on chronic pelvic pain and health-related quality of life. Int J Gynaecol Obstet. 2014;124:139–42. 24225262 10.1016/j.ijgo.2013.07.029

[12] Sandall J , Tribe RM , Avery L , Mola G , Visser GH , Homer CS , et al. Short-term and long-term effects of caesarean section on the health of women and children. Lancet. 2018;392:1349–57. 30322585 10.1016/S0140-6736(18)31930-5

[13] Slabuszewska-Jozwiak A , Szymanski JK , Ciebiera M , Sarecka-Hujar B , Jakiel G . Pediatrics consequences of caesarean section-a systematic review and meta-analysis. Int J Environ Res Public Health. 2020;17:8031. 33142727 10.3390/ijerph17218031 PMC7662709

[14] Li Y , Sjolander A , Song H , Cnattingius S , Fang F , Yang Q , et al. Associations of parental and perinatal factors with subsequent risk of stress-related disorders: a nationwide cohort study with sibling comparison. Mol Psychiatry. 2022;27:1712–9. 34974524 10.1038/s41380-021-01406-5 PMC9095463

[15] Kawamoto T , Nitta H , Murata K , Toda E , Tsukamoto N , Hasegawa M , et al. Rationale and study design of the Japan environment and children’s study (JECS). BMC Public Health. 2014;14:25. 24410977 10.1186/1471-2458-14-25 PMC3893509

[16] Michikawa T , Nitta H , Nakayama SF , Yamazaki S , Isobe T , Tamura K , et al. Baseline profile of participants in the Japan environment and Children’s study (JECS). J Epidemiol. 2018;28:99–104. 29093304 10.2188/jea.JE20170018 PMC5792233

[17] Araki A , Kanemastu M , Yokosawa S , Arayashiki R , Aizumi I , Hujishima K. A study for developing parenting stress-short form scale [in Japanese]. J Child Health. 2005;64:408–16.

[18] Narama M , Kanematsu Y , Araki A , Maru M , Nakamura N , Takeda J , et al. Validity and reliability of the Japanese version of the parenting stress index. J Child Health. 1999;58:610–6.

[19] Kanematsu Y , Araki A , Narama M , Shirahata N , Maru M , Arayashiki R . PSI-SF parenting stress index manual. 2nd ed. Tokyo: Koyou mondai kenkyukai; 2015.

[20] Hatakeyama T , Matsumura K , Tsuchida A , Inadera H , Group JECS . Factor structure of the parenting stress index-short form used in the Japan environment and Children’s study. Sci Rep. 2022;12:19123. 36352189 10.1038/s41598-022-23849-8 PMC9646740

[21] Matsumura K , Hamazaki K , Tsuchida A , Kasamatsu H , Inadera H , Group JECS . Education level and risk of postpartum depression: results from the Japan environment and children’s study (JECS). BMC Psychiatry. 2019;19:419. 31882000 10.1186/s12888-019-2401-3 PMC6935197

[22] Craig CL , Marshall AL , Sjöström M , Bauman AE , Booth ML , Ainsworth BE , et al. International physical activity questionnaire: 12-country reliability and validity. Med Sci Sports Exerc. 2003;35:1381–95. 12900694 10.1249/01.MSS.0000078924.61453.FB

[23] Murase N , Katsumura T , Ueda C , Inoue S , Shimomitsu T . Validity and reliability of Japanese version of international physical activity questionnaire. J Health Welf Stat. 2002;49:1–9.

[24] Matsumura K , Hamazaki K , Tsuchida A , Kasamatsu H , Inadera H , JECS Group. Causal model of the association of social support during pregnancy with a perinatal and postpartum depressive state: a nationwide birth cohort - the Japan environment and children’s study. J Affect Disord. 2022;300:540–50. 34979183 10.1016/j.jad.2021.12.117

[25] Furukawa TA , Kawakami N , Saitoh M , Ono Y , Nakane Y , Nakamura Y , et al. The performance of the Japanese version of the K6 and K10 in the world mental health survey Japan. Int J Methods Psychiatr Res. 2008;17:152–8. 18763695 10.1002/mpr.257 PMC6878390

[26] Sakurai K , Nishi A , Kondo K , Yanagida K , Kawakami N. Screening performance of K6/K10 and other screening instruments for mood and anxiety disorders in Japan. Psychiatry Clin Neurosci. 2011;65:434–41. 21851452 10.1111/j.1440-1819.2011.02236.x

[27] Kessler RC , Andrews G , Colpe LJ , Hiripi E , Mroczek DK , Normand SL , et al. Short screening scales to monitor population prevalences and trends in non-specific psychological distress. Psychol Med. 2002;32:959–76. 12214795 10.1017/s0033291702006074

[28] Matsumura K , Hamazaki K , Tsuchida A , Inadera H , JECS Group. Omega-3 fatty acid intake during pregnancy and risk of infant maltreatment: a nationwide birth cohort - the Japan environment and children’s study. Psychol Med. 2021:1–10. DOI: 10.1017/S0033291721002427. PMC9975990 34176535

[29] Nakamura M , Matsumura K , Ohnuma Y , Yoshida T , Tsuchida A , Hamazaki K , et al. Association of cesarean birth with prevalence of functional constipation in toddlers at 3 years of age: results from the Japan environment and Children’s study (JECS). BMC Pediatr. 2021;21:419. 34556067 10.1186/s12887-021-02885-9 PMC8459474

[30] Dinan TG , Kennedy PJ , Morais LH , Murphy A , Long-Smith CM , Moloney GM , et al. Altered stress responses in adults born by caesarean section. Neurobiol Stress. 2022;16:100425. 35024387 10.1016/j.ynstr.2021.100425 PMC8733342

[31] Lagercrantz H. The good stress of being born. Acta Paediatr. 2016;105:1413–6. 27870197 10.1111/apa.13615

[32] Rieder R , Wisniewski PJ , Alderman BL , Campbell SC . Microbes and mental health: a review. Brain Behav Immun. 2017;66:9–17. 28131791 10.1016/j.bbi.2017.01.016

[33] Zhang T , Brander G , Mantel A , Kuja-Halkola R , Stephansson O , Chang Z , et al. Assessment of cesarean delivery and neurodevelopmental and psychiatric disorders in the children of a population-based Swedish birth cohort. JAMA Netw Open. 2021;4:e210837. 33666663 10.1001/jamanetworkopen.2021.0837 PMC7936261

[34] Yuda M . Public and social environment changes and caesarean section delivery choice in Japan. BMC Res Notes. 2018;11:633. 30176901 10.1186/s13104-018-3746-2 PMC6122532

[35] Zhang J , Liu Y , Meikle S , Zheng J , Sun W , Li Z. Cesarean delivery on maternal request in Southeast China. Obstet Gynecol. 2008;111:1077–82. 18448738 10.1097/AOG.0b013e31816e349e

[36] Zanardo V , Giliberti L , Giliberti E , Volpe F , Straface G , Greco P. The role of elective and emergency cesarean delivery in maternal postpartum anhedonia, anxiety, and depression. Int J Gynaecol Obstet. 2018;143:374–8. 30159888 10.1002/ijgo.12657

