# Supplementary material for: Cesarean section and parenting stress: Results from the Japan Environment and Children’s Study
Source: Eur Psychiatry. 2023 Jan 24;66(1):e18. doi: 10.1192/j.eurpsy.2023.5 (PMC9970147; doi:10.1192/j.eurpsy.2023.5)
Supplement: Supplementary file 1 [file S0924933823000056sup001.docx]

**Supplementary Table 1.** Overview of the Japanese 19-item version of the Parenting Stress Index Short Form used in the Japan Environment and Children’s Study.

| Item |  | Strongly disagree | Dis-agree | Not sure | Agree | Strongly agree |
| --- | --- | --- | --- | --- | --- | --- |
| 1. | I enjoy being a parent | ▢ | ▢ | ▢ | ▢ | ▢ |
| 2. | When caring for my child is difficult, I resort to asking for help or advice | ▢ | ▢ | ▢ | ▢ | ▢ |
| 3. | My child is active to the extent of overwhelming me | ▢ | ▢ | ▢ | ▢ | ▢ |
| 4. | My child has difficulty focusing attention | ▢ | ▢ | ▢ | ▢ | ▢ |
| 5. | My child rarely does things that make me feel good | ▢ | ▢ | ▢ | ▢ | ▢ |
| 6. | My child cries or fusses rather often | ▢ | ▢ | ▢ | ▢ | ▢ |
| 7. | My child does not smile very much, unlike most children | ▢ | ▢ | ▢ | ▢ | ▢ |
| 8. | My child does some things that bother me a lot | ▢ | ▢ | ▢ | ▢ | ▢ |
| 9. | My child is easily upset by small things | ▢ | ▢ | ▢ | ▢ | ▢ |
| 10. | My child imposes demands on me more than most children | ▢ | ▢ | ▢ | ▢ | ▢ |
| 11. | My child is always attached to me | ▢ | ▢ | ▢ | ▢ | ▢ |
| 12. | I do not feel able to handle things very well | ▢ | ▢ | ▢ | ▢ | ▢ |
| 13. | Since giving birth to my child, I feel unable to do most of the things I like to do | ▢ | ▢ | ▢ | ▢ | ▢ |
| 14. | I feel that it is my fault every time my child does something wrong | ▢ | ▢ | ▢ | ▢ | ▢ |
| 15. | My spouse does not give me as much help as I expected | ▢ | ▢ | ▢ | ▢ | ▢ |
| 16. | Having a child has created more problems with my spouse | ▢ | ▢ | ▢ | ▢ | ▢ |
| 17. | I feel alone with no friends | ▢ | ▢ | ▢ | ▢ | ▢ |
| 18. | I have been experiencing more illness, aches, and pains over the past six months | ▢ | ▢ | ▢ | ▢ | ▢ |
| 19. | I am unable to enjoy things as I used to | ▢ | ▢ | ▢ | ▢ | ▢ |

Scores are calculated as follows.

For items 3-19: 1 = Strongly disagree , 2 = Disagree, 3 = Not sure, 4 = Agree, 5 = Strongly agree.

For items 1 and 2 (with reversed valence): 5 = Strongly disagree, 4 = Disagree, 3 = Not sure, 2 = Agree, 1 = Strongly agree.

Difficult child factor: items 3, 4, 6, 8, 9, 10, and 11.

Parental distress factor: items 1, 2, 12, 13, 14, 17, 18, and 19.

Spouse factor: items 15 and 16.

Not belonging to any factor: items 5 and 7.
